# "Open Sesame" to the complexity of pattern recognition receptors of myeloid-derived suppressor cells in cancer

**DOI:** 10.3389/fimmu.2023.1130060

**Published:** 2023-02-22

**Authors:** Tian Wang, Yushu Hu, Silvia Dusi, Fang Qi, Silvia Sartoris, Stefano Ugel, Francesco De Sanctis

**Affiliations:** Department of Medicine, Section of Immunology, University of Verona, Verona, Italy

**Keywords:** PRR, MDSC, TLRs, NLRs, cancer, immune therapy

## Abstract

Pattern recognition receptors are primitive sensors that arouse a preconfigured immune response to broad stimuli, including nonself pathogen-associated and autologous damage-associated molecular pattern molecules. These receptors are mainly expressed by innate myeloid cells, including granulocytes, monocytes, macrophages, and dendritic cells. Recent investigations have revealed new insights into these receptors as key players not only in triggering inflammation processes against pathogen invasion but also in mediating immune suppression in specific pathological states, including cancer. Myeloid-derived suppressor cells are preferentially expanded in many pathological conditions. This heterogeneous cell population includes immunosuppressive myeloid cells that are thought to be associated with poor prognosis and impaired response to immune therapies in various cancers. Identification of pattern recognition receptors and their ligands increases the understanding of immune-activating and immune-suppressive myeloid cell functions and sheds light on myeloid-derived suppressor cell differences from cognate granulocytes and monocytes in healthy conditions. This review summarizes the different expression, ligand recognition, signaling pathways, and cancer relations and identifies Toll-like receptors as potential new targets on myeloid-derived suppressor cells in cancer, which might help us to decipher the instruction codes for reverting suppressive myeloid cells toward an antitumor phenotype.

## Introduction

Myeloid-derived suppressor cells (MDSCs) have recently emerged as key players in regulating host immune responses in many pathologies. Although this heterogeneous population of myeloid cells was identified in many tumor histotypes, its relevance in orchestrating innate and adaptive immune responses has also been ascribed in infectious diseases, such as sepsis (1) and COVID-19 (2, 3), as well as in pregnancy (4, 5). MDSCs include two main subsets of myeloid cells—monocytic (M-MDSC) and polymorphonuclear (PMN-MDSC). Although phenotypically these immune regulators resemble normal monocyte and polymorphonuclear counterparts, MDSCs are endowed with peculiar molecular, metabolic, and immune suppressive features toward natural killer (NK), T and B lymphocyte activation and proliferation (6). In mice, M-MDSCs and PMN-MDSCs cannot be distinguished from inflammatory monocytes and granulocytes/polymorphonuclear leukocytes (PMNs) according to surface markers (M-MDSC: CD11b^+^ Ly6C^hi^ cells; PMN-MDSC: CD11b^+^ Ly6C^int^ Ly6G^+^cells) (7). In humans, three main MDSC subsets have been identified: M-MDSCs and PMN-MDSCs are defined as CD11b^+^ CD14^+^ CD15^-^ CD33^+^ HLA-DR^-^ and CD11b^+^ CD14^-^ CD15^+^ (or CD66b^+^) CD33^+^-expressing cells, respectively. Recently a third MDSC population, named early-stage MDSCs (eMDSCs), which is negative for CD14, CD15, and HLA-DR markers and expresses CD33, was identified and represented a small cell subset of myeloid precursors (7). These days, PMN-MDSCs can be separated from neutrophils in human peripheral blood samples by employing gradient separation or novel disclosed markers such as lectin-type oxidized LDL receptor 1 (LOX-1), whereas M-MDSCs are characterized by the low expression of HLA-DR, which is instead highly present in monocytes (7).

Despite these common features, several studies have shown that specific immunological contexts define the direction of myelopoietic routes toward the generation of PMNs, monocytes, or their immunosuppressive counterparts. Indeed myeloid cells patrol immune cells, promptly reaching the infection site to fight pathogen spreading by many mechanisms, including phagocytosis, killing, neutralization, inflammation, priming, and support of adaptive immune responses. A pathogen infection results in the fast mobilization of both PMNs and monocytes toward the inflammation site in an attempt to defeat pathogen dissemination. Classical myeloid cell activation occurs through sensing a broad spectrum of danger-associated molecular patterns (DAMPs) and pathogen-associated molecular patterns (PAMPs) by their pattern recognition receptors (PRRs). PRRs are composed of several parts and include ligand recognition domains, intermediate domains, and effector domains. After recognizing and binding to their respective ligands, they recruit adaptor molecules and initiate downstream signaling pathways such as cell activation and transcription of inflammatory genes (8). PAMPs can be expressed by pathogens or invasive microbes, whereas DAMPs are stress signals released or exposed on the membrane of damaged cells and include components that are normally found intracellularly, such as adenosine triphosphate, calreticulin, annexin A1, high mobility group box 1 (HMGB1), heat shock (HSP), and S100 proteins (9). Dr. William Coley, acknowledged as the forefather of cancer immune therapy, provided in 1891 the first evidence that bacterial immune toxins could reactivate the host immune system to fight tumor progression in inoperable cancer patients, without knowing either the mechanism of action of those PAMPs or the target receptors and immune cells (10). Recently, we demonstrated that hyperthermic-mediated secondary apoptosis of tumor cells induces the release of HMGB1, which, in turn, supports the activation of dendritic cells and sustains the activation of a tumor-specific adaptive immune response that restricts prostate cancer outgrowth (11). Thus, DAMPs can protect the host by supporting immunogenic cell death, thus alerting the immune system to recognize tumor cell components. However, chronic DAMP stimulation in sterile inflammatory conditions, sustained directly (actively secreted molecules) or indirectly (because of cell death) by tumors, shows opposite effects by supporting the recruitment and immunosuppressive functions of MDSCs and TAMs, contributing to cancer cell survival, local invasion, and metastatic dissemination in many neoplasias (12). Injured or stressed cancer cells release redundant DAMPs that potently work on specific PRRs on MDSCs (13). Moreover, the linkage between specific PRRs and cancer-associated microorganisms is well documented, including *Helicobacter pylori* (14) and Epstein–Barr virus (15) in gastric cancer, hepatitis B virus and hepatitis C virus in hepatocellular carcinoma, human papillomavirus (16) in cervical cancer, and dysbiotic gut microorganisms (for example, *Bacteroides fragilis*) (17) in pancreatic cancer and colorectal carcinoma (CRC) (18). The immune contexture, timing, and concentration of DAMPs and PAMPs can altogether orchestrate different effects on the host immune system, participating in defining the fate of cancer therapy. Chronic exposure to inflammatory stimuli usually associated with unresolved infections and chronic inflammation, cancer included, sustains a pathological deviation of host myelopoiesis with increased dysfunctional monocytes and PMNs. MDSC expansion and acquisition of immune suppressive abilities take place through sequential events that occur in different microenvironments. In the first phase, chronic stimulation by granulocyte colony-stimulating factor (G-CSF), macrophage colony-stimulating factor (M-CSF), and granulocyte macrophage colony-stimulating factor (GM-CSF) sustains MDSC generation and expansion in the bone marrow. Those cells are then mobilized in the periphery and can acquire more pronounced immune suppressive features if exposed to a specific microenvironment, such as in the tumor core, by vascular endothelial growth factor, interleukins, or a hypoxic state (5). Activation of signal transducer and activator of transcription 3 (STAT3), CCAAT/enhancer binding protein β (C/EBPβ) (19), and nuclear factor kappa-light-chain-enhancer of activated B cells (NF-κB) (20) signaling is crucially involved in both stages. Although many cytokines can promote pathological hematopoiesis deviation, recent studies underlined that MDSC generation and suppression can be recapitulated *in vitro* by the combination of GM-CSF + IL6 and GM-CSF + G-CSF in mouse and human bone marrow progenitors, respectively (19). Cancer sustains this chronic inflammation to corrupt host hematopoiesis toward the generation and recruitment of immune regulatory cells, including MDSCs. These cells can support tumor progression and metastatic spreading by hijacking cancer immune surveillance by restricting T cell entrance into the tumor and by establishing a hostile tumor microenvironment (TME) impairing the fitness of cytotoxic effectors (21). Moreover, MDSCs can directly support cancer progression by promoting epithelial–mesenchymal transition and tumor stemness and establishing a microenvironment suitable for seeding metastatic tumor cells, named the premetastatic niche (22–24). Many studies have associated MDSC expansion with worse patient clinical outcomes in solid tumors, including skin (25), pancreatic (26), brain (27), prostate (28), lung (29), and breast cancers (30, 31), and have uncovered MDSC enumeration as a prognostic biomarker of response to immune therapies (32, 33). Thus, deciphering MDSC ontogeny and selective targeting strategies remain as cornerstones for overcoming the current limitations of cancer immunotherapies.

It has become apparent that, in many cancer types, PRRs play a central role in modulating a vast array of tumor-inhibiting and tumor-promoting cellular responses both in immune cells within the tumor microenvironment and directly in cancer cells. MDSCs share with physiological myeloid cells the expression of many Toll-like receptors (TLRs), including TLR2 and TLR7-8, in both mice and humans (34, 35). Thus, TLR agonists/antagonists were recently employed in both preclinical and translational research to repolarize myeloid cells (including MDSCs) and support the activation and efficacy of adaptive antitumor immunity. Whether PRRs are differentially expressed in MDSCs, monocytes, and PMNs and whether their triggering activates different signaling pathways are still open questions. In this review, we discuss the expression of PRRs on MDSCs, how PRRs contribute to MDSC development and function, the signaling pathways included, and the targeting approaches, with the final aim of unveiling the promise of potential agonists to target myeloid immune regulatory functions and to restore host cancer immune surveillance.

## PRR expression on MDSCs

**Figure 1:**
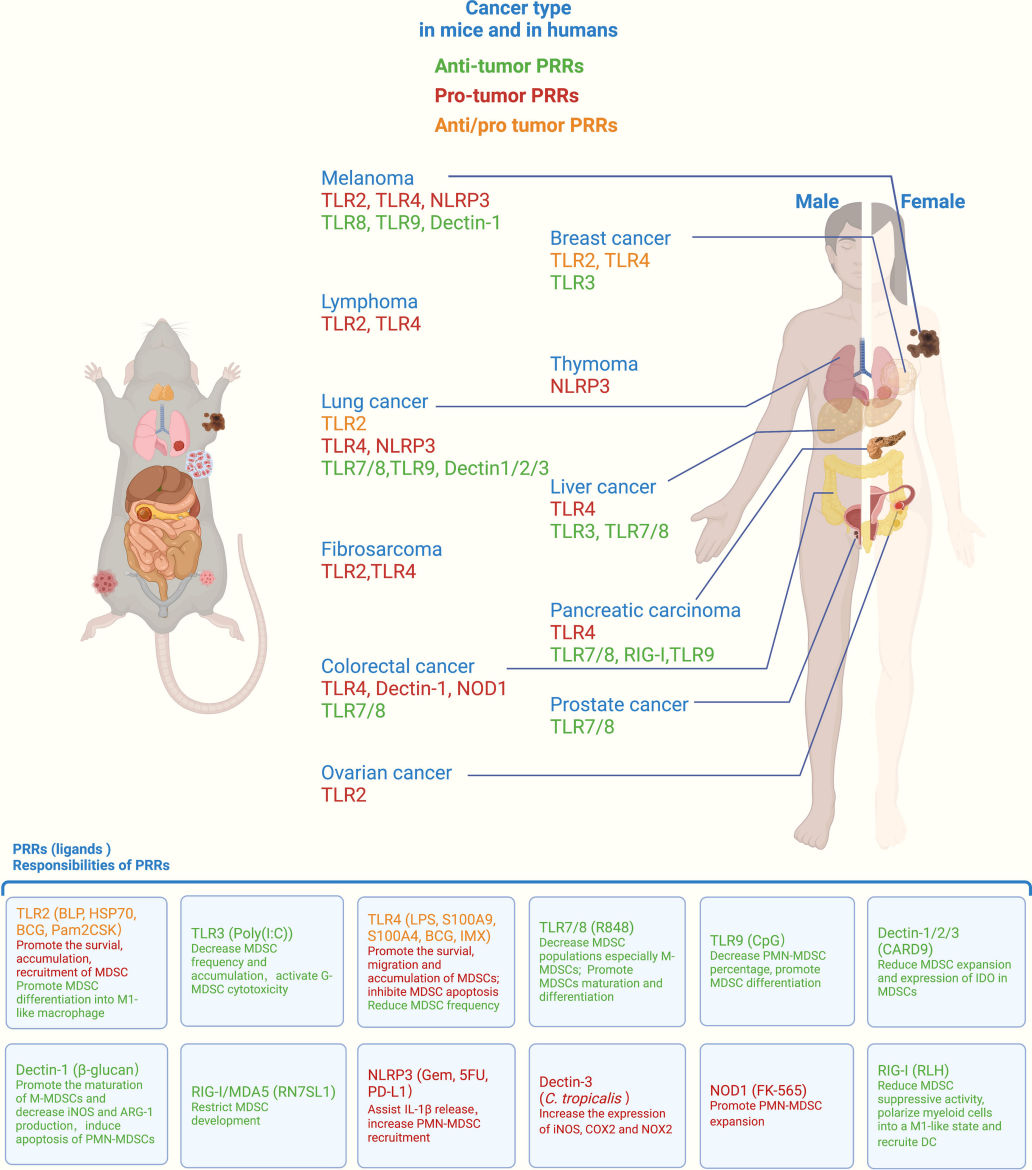
Roles of PRRs in orchestrating myeloid-derived suppressor cell (MDSC) function across different cancers. PRRs represent the primary MDSC receptors for integrating signals from pathogens or damaged cells. By recognizing different ligands, PRRs orchestrate MDSC immunosuppressive function, survival, migration, accumulation, differentiation, and soluble molecule release, thus exerting protumor or antitumor effects in mice and humans. PRRs, pattern recognition receptors; TLR, Toll-like receptor; NLRP3, NLR family pyrin domain containing 3; NOD1, nucleotide oligomerization domain; RIG-I, retinoic acid-inducible gene-I; BLP, bacterial lipoprotein; HSP70, heat shock protein 70; BCG, Bacille–Calmette–Guerín; LPS, lipopolysaccharide; S100A9, S100 calcium-binding protein A9; CARD9, caspase activation and recruitment domain 9; Gem, gemcitabine; 5FU, 5-fluorouracil; PD-L1, programmed death-ligand 1; RLH, RIG-I-like helicases. All listed PRRs were demonstrated to regulate MDSC function in mice, whereas only the PRRs indicated with lines were proven to modulate MDSC function in human cancers. The role of ALRs is not shown above due to the lack of relevant reports.

In the 1990s, the hypothesis of PAMPs and their specific receptors was proposed by Janeway, which was of epoch-making significance and changed research on innate immunity (36). TLRs are one of the earliest PRRs discovered in the innate immune system, and TLR4 can induce the activation of NF-κB and the expression of the costimulatory molecules CD80 and CD86, giving indispensable second signals for T lymphocyte activation (37, 38). After the discovery of TLR4, many PRRs and their corresponding ligands were discovered. PRRs can be classified into five groups based on protein domain homology: TLRs, nucleotide oligomerization domain (NOD)-like receptors (NLRs), retinoic acid-inducible gene-I (RIG-I)-like receptors (RLRs), C-type lectin receptors (CLRs), and absent in melanoma-2 (AIM2)-like receptors (ALRs) (7, 39). With the advances in studying PRRs’ structure, function, and distribution, the role of PRRs in arranging MDSC function in different types of cancer has become clearer, as summarized in **Figure 1**.

### Toll-like receptors

TLRs are the first family of PRRs to be discovered and defined. In total, 13 types of TLRs (TLR1–10 in humans and TLR1–9 and TLR 11–13 in mice) have been identified. All TLRs are type I transmembrane glycoproteins and share a similar domain organization: an extracellular N-terminal ectodomain contains leucine-rich repeats (LRRs, responsible for recognition of DAMPs and PAMPs), a single transmembrane region, and an intracellular cytosolic Toll/IL-1R (TIR) region that is responsible for signal transduction (8, 40). TLRs localize in different positions of the cells. TLR1, 2, 4, 5, 6, and 10 are exposed on the cell surface, usually in the form of heterodimers or homodimers, and recognize pathogen membrane components, such as lipids, peptidoglycans, lipoproteins, or DAMPs, such as HSP. TLR3, TLR7, TLR8, and TLR9 are expressed in the form of homodimers on endosomal membranes and recognize intracellular RNA or DNA from microorganisms or damaged host cells. In monocytes and neutrophils, the activation of TLRs results in an inflammatory response by producing and secreting a variety of proinflammatory and antiviral factors (41). The consequence of different TLR activation in MDSCs in cancer is controversial and possibly context dependent. In general, cell surface TLR activation contributes more to MDSC suppressive function and a pro-tumor effect, yet endosomal TLR activation mainly decreases the ability of MDSCs and exerts an antitumor effect (42, 43). Cell surface TLRs, including TLR1, TLR2, TLR4, TLR5, TLR6, and TLR10, can actively capture extracellular information, including tumor cell-released DAMPs or apoptotic cancer cell fragments, such as HMGB1, HSP, and S100, as well as extracellular vesicles containing changed cell components. The corresponding signal transduction will amplify the effect of the immune-suppressive response by producing a variety of proteins, including arginase 1 (ARG1), inducible nitric oxide synthase (iNOS, also named NOS2), indoleamine 2,3-dioxygenase 1 (IDO1), prostaglandin E2 (PGE_2_), programmed death-ligand 1 (PD-L1), CD40, tumor necrosis factor (TNF), IL-1β, IL-6, cyclooxygenase-2 (COX2), *etc.* Thus, creating an immunosuppressive TME with less antigen presentation hampers T cell or NK cell activation and proliferation and reduces cytotoxicity (44). Intracellular TLRs, including TLR3, TLR7, TLR8, and TLR9, are capable of detecting intracellular nucleic acids and inducing the production of type I IFN (IFN1), which plays a role in antitumor immunity by diverse mechanisms, including reducing ARG1 and NOS2 production and improving the survival of cytotoxic lymphocytes.

TLR2 usually forms heterodimers with TLR1 or TLR6 to recognize ligands, including bacterial lipoteichoic acid, triacyl lipopeptide, arabinomannan, peptidoglycan, pore protein, and fungal zymosan. In MDSCs, the recognition pattern is changed. TLR1/2 exhibit duality in regulating MDSC behavior during tumor progression. Indeed TLR2 activation with bacterial lipoproteins in MDSCs supports cancer growth by promoting MDSC survival, accumulation, and recruitment to the tumor microenvironment in mouse lymphoma, melanoma, lung cancer, fibrosarcoma, and CRC models and is verified to be correlated with worse prognosis in human CRC tissues (45–47). However, an opposite correlation was found by other groups who showed that TLR1/2 expression and activation were associated with better prognosis in lung cancer patients, which was further confirmed in mouse lung cancer and breast cancer models by promoting MDSC differentiation into M1 macrophages (48, 49). In tuberculosis, which is associated with a higher lung cancer risk (50), *Mycobacterium bovis* Bacille–Calmette–Guerín (BCG) infection induces TLR2 and TLR4 expression in mouse MDSCs, further upregulating CD40, PD-L1, and CD69 expression in both G-MDSCs and M-MDSCs as well as iNOS expression, leading to increased nitric oxide (NO) production required for the suppression of T cell proliferation in BCG-infected mice (51). However, hematopoietic stem cells exposed to BCG showed stronger anti-tuberculosis immune protection when engrafted in a new host by producing “trained” monocytes/macrophages (52). These findings challenge the current vision of adaptive immunity as the only immune system components endowed with enhanced functional capacity. Several recent studies have demonstrated further evidence of memory-like or stimulus-imprinted innate immune education through PAMP-PRR recognition in PMNs (53, 54). In CRC patients, increased circulating MDSCs were found in peripheral blood as well as accumulated in tumor tissues, and this process was driven by S100 calcium-binding protein A9 (S100A9) *via* TLR4-mediated NF-κB signaling pathways in MDSCs (55). C-X-C motif chemokine ligand 10 (CXCL10) plays an important role in hepatic disease. Recently, it was reported that CXCL10 contributes to hepatocellular apoptosis through TLR4 on MDSCs instead of its common receptor—CXC motif receptor 3—in patients with hepatocellular carcinoma (HCC), thus leading to cancer recurrence. Knockout or inhibition of CXCL10/TLR4 significantly reduced the M-MDSC levels and constrained tumor growth in a mouse recurrent tumor model (56). S100A4 can also bind to TLR4 on MDSCs in fibrosarcoma-, melanoma-, and lung cancer-bearing mouse models (57). The soluble protein involved in pancreatic ductal adenocarcinoma (PDAC) tumorigenesis—named pancreatic adenocarcinoma upregulated factor—binds to TLR4 on MDSCs and triggers the production of ARG1, NO, and reactive oxygen species (ROS) in pancreatic tumor-bearing mice (58). Soluble calreticulin has also been demonstrated to be involved in the migration and survival process of tumor-derived MDSCs *via* interaction with TLR4 (59). Notably, TLR4 activation in MDSCs [activated by BCG (60) and Immunomax (61)] reduced the MDSC frequency and thereby suppressed rat bladder cancer progression or mouse breast cancer metastasis, respectively.

Nevertheless, LPS-activated TLR4 has been proven to expand MDSCs *in vivo.* Recently, it has aroused interest again on its long-term functional “training” on myeloid cells in the pathogenesis of chronic inflammatory diseases (62, 63), which goes along with extensive long-lasting epigenetic memory in hematopoietic stem cells, provoking drastic changes in the landscape of substream myeloid progenitor cells (64). An increased transcriptional response of open myeloid enhancers to secondary stimulation was observed to help defend against secondary infection from gram-negative bacteria.

Endosome-localized TLR3, TLR7/8, and TLR9 are typical nucleotide sensors. TLR3 and TLR9 agonists reduce MDSCs in an EG7 lymphoma mouse model (65). TLR3 activates G-MDSC cytotoxicity and inhibits tumor growth through the production of ROS/reactive nitrogen species (RNS) (66).

TLR7/8 and TLR9 agonists have been successfully used in cancer treatment. In CRC mouse models, applying the TLR9 ligand CpG decreased the PMN-MDSC percentage, prevented MDSC suppressive functions on T cell proliferation, and promoted their differentiation into mature myeloid cells with enhanced expression of the macrophage differentiation marker F4/80, the DC maturation markers MHCII and CD11c, and the costimulatory molecule CD80 (67). CpG also decreased the percentage of MDSCs in the spleen and inhibited their infiltration into mouse B16 melanoma tumors (68). The TLR7/8 agonist resiquimod (R848) decreases both intratumoral and circulating MDSC populations, especially M-MDSCs, with up to fivefold decrease in the tumor. R848 promoted MDSC maturation and differentiation with upregulation of the surface molecules CD11c, F4/80, MHC-I, and MHC-II (69). Similarly, TLR7 agonists reduced MDSCs by 50% and nearly doubled the M1/M2 ratio to shift the TME toward a more inflammatory state, thus enhancing the efficacy of CAR-T therapy in 4T1 breast cancer models (70).

### NOD-like receptors

Unlike TLRs localized on the cell membrane, NLRs are intracellular PRRs consisting of three binding domains: central NOD (nucleotide-binding and oligomerization domain), N-terminal effector domain, and C-terminal LRRs. According to the different N-terminal domains, NLRs are divided into four subgroups: the acidic transactivation domain, the baculoviral inhibitory repeat-like domain, the caspase activation and recruitment domain (CARD; NLRC), and the pyrin domain (NLRP) (71). Sensing of danger signals by NLRs leads to their oligomerization into large macromolecular scaffolds and the rapid deployment of effector signaling cascades to restore homeostasis (72). NLR activation in MDSCs is closely related to colitis-associated cancer. NOD1 (a main member of the NLRC) activation by FK-565 arouses systemic inflammation, with expanded PMN-MDSCs, followed by the later recruitment of M-MDSCs into the peritoneal compartment, and increases the expression of ARG1 to exert immunosuppressive function, thus driving carcinogenesis toward CRC (73). Some recent studies have also confirmed the key role of NLRP3 in MDSC promotion of tumor progression. Some chemotherapeutic agents, gemcitabine (Gem) and 5-fluorouracil, were also proven to induce the expression of IL-1β in an NLRP3-dependent manner in MDSCs, thus curtailing anticancer immunity (74). NLRP3 inhibition diminished PMN-MDSC recruitment in response to anti-PD-1 Ab therapy in a mouse melanoma model (75, 76), and NLRP3 inflammasome blockade reduced the frequencies of MDSCs in the tumor tissues, spleen, and peripheral blood in lymphoma and decreased the expression of immunosuppressive genes (Pdcd1l1, Arg1, Il10, and Tgfb1) in PMN-MDSCs isolated from tumor-bearing mice (77, 78). In addition, NLRP3-deficient MDSCs (isolated from knockout tumor-bearing mice) are less efficient in reaching the tumor site (79).

### RIG-I-like receptors

Retinoic acid-inducible gene 1 executes its antiviral activity by recognizing viral RNA and releasing interferon-beta and other proinflammatory cytokines (8). RIG-I-like receptors are intracellular PRRs. RLRs include several molecules, such as retinoic acid-inducible gene I, melanoma differentiation-associated protein 5 (MDA5), and LGP2 (80). Mimicking viral infections with immune-activating RNA species is a potential strategy for tumor immunotherapy because of the shared fundamental features of immune responses against viruses and tumors. RIG-I-like helicase triggering induces an IFN-driven immune response and reprograms the TME of pancreatic cancer by reducing MDSC suppressive activity, polarizing myeloid cells into an M1-like state and recruiting DCs (81). Since the low rate of tumor-infiltrating immune cells, the development of lymphocyte exhaustion, and antigen insufficiency are common mechanisms that limit chimeric antigen receptor (CAR)-T cell therapeutic effectiveness (82), CAR-T cells engineered to deliver RIG-I/MDA5 agonists were able to restrict MDSC development by reducing their release of anti-inflammatory cytokines (*e*.*g*., TGF-β) and fostered DC subsets with costimulatory features, thus enhancing CAR-T cell efficacy in B16 melanoma-bearing mice, even when heterogeneous CAR antigen tumors lack adequate neoantigens (83).

### C-type lectin receptors

CLRs are a class of transmembrane phagocytic PRRs. They play a role in both the innate immune system and acquired immunity. It recognizes and binds to PAMPs and places pathogens in cytoplasmic vesicles for direct digestion and elimination to control the infection (84). CLRs include three members: Dectin-1, Dectin-2, and Dectin-3 (8). A study indicated that LOX-1 (member of the Dectin-1 subgroup) was a specific marker for human PMN-MDSCs since LOX-1 could not be detected in the neutrophils of healthy donors, whereas LOX-1^+^ neutrophils (5–15% in cancer patients and 15–50% in tumor tissues) had potent immune suppressive activity and other biochemical characteristics of PMN-MDSCs (85). *C. tropicalis* facilitates the immunosuppressive function of MDSCs by increasing the expression of iNOS, COX2, and NOX2 and the production of NO and ROS *via* C-type lectin receptor Dectin-3-dependent pathways. NO produced by MDSCs enhanced aerobic glycolysis. Furthermore, *C. tropicalis* upregulates the expression of HIF-1α target genes encoding the glycolytic enzymes GLUT1, HK2, PKM2, LDHA, and PDK1 in aerobic glycolysis, which drives colorectal cancer (86). The CLR downstream signaling molecule adapter protein CARD9, which is highly expressed in myeloid cells, was proven to reduce MDSC expansion and the expression of indoleamine 2,3-dioxygenase in MDSCs to slow down the lung cancer progression (87).

Fungal-derived polysaccharide β-glucan (a major ligand of Dectin-1) induces the differentiation of M-MDSCs (monocytic MDSCs) into a more mature population with a CD11c^+^ F4/80^+^ phenotype and drastically decreases iNOS and ARG-1 production *via* the dectin-1 pathway *in vitro*, thereby leading to delayed tumor progression in a mouse Lewis lung cancer model (88). Another study also demonstrated that β-glucan induced subsequent apoptosis in PMN-MDSCs while converting M-MDSCs to potent antigen-presenting cells, which could promote antigen-specific CD4 and CD8 T cell responses (89). Furthermore, recent reports have shown a novel and profound effect of β-glucan in promoting a sustained and enhanced response of myeloid cells to secondary infectious or inflammatory challenges as a representation of trained innate immunity (90, 91). Nevertheless, β-glucan training teaches hematopoietic progenitors toward better control of melanoma and lung cancer progression. Indeed β-glucan stimulation caused transcriptomic and epigenetic rewiring of granulopoiesis toward an antitumor phenotype. This imprinting was carried for a long term in adoptively transferred neutrophils from β-glucan-trained mice to naive recipients and suppressed tumor growth in a ROS-dependent manner, giving us new insight into antitumor immunity regulation through the induction of trained immunity (92).

## Signaling pathways in PRRs of MDSCs

**Figure 2:**
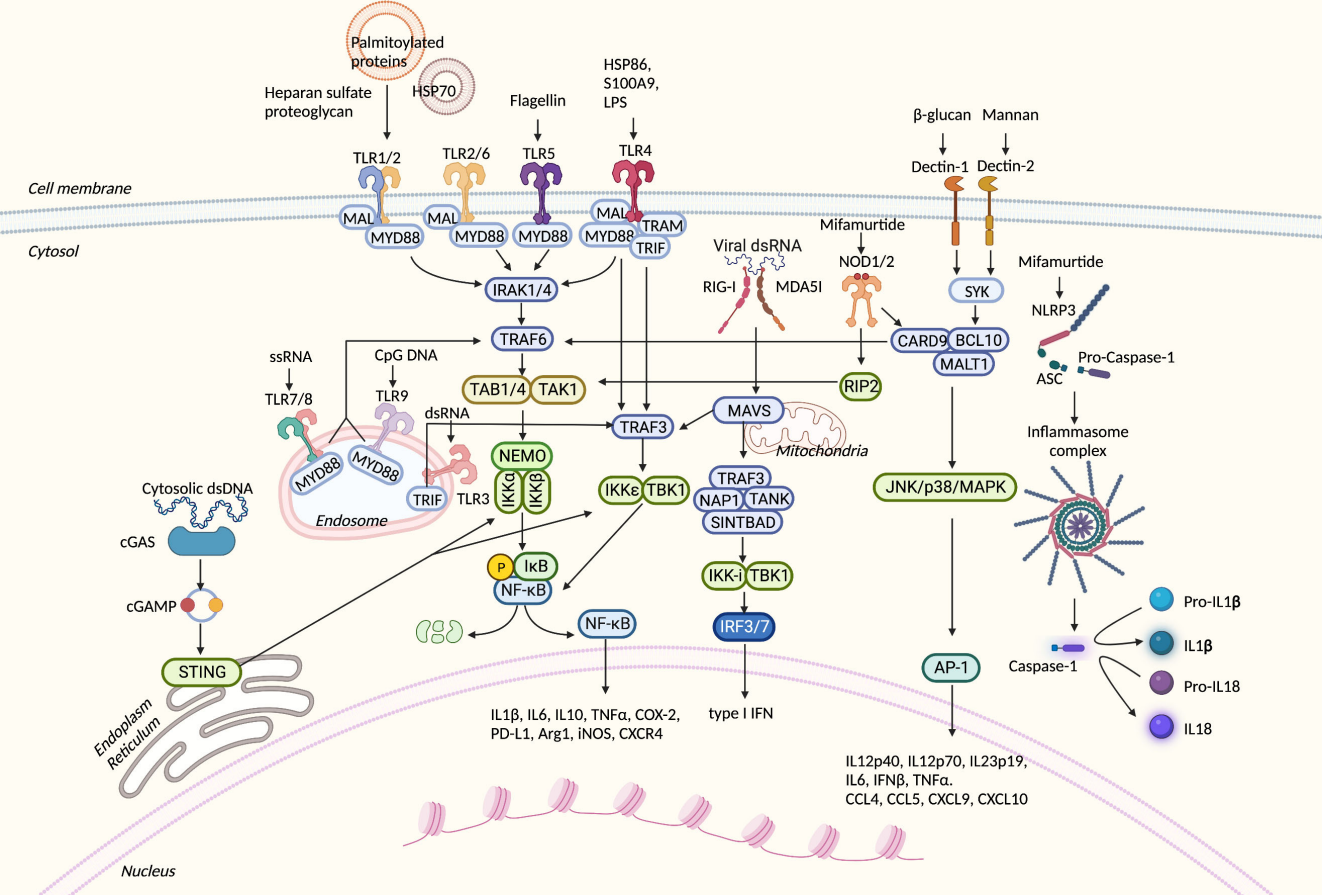
Overview of PRR-related signaling pathways in myeloid-derived suppressor cells (MDSCs). PRRs can sense numerous danger signals released by pathogens and damaged normal and neoplastic cells. After binding to proper ligands, PRRs recruit adaptor proteins and trigger downstream signal transduction. These processes result in the translocation of transcription factors into the nucleus, expression of inflammatory-related genes, or direct cytokine activation from their inactive forms. PRRs, pattern recognition receptors; TLR, Toll-like receptor; HSP70, heat shock protein 70; LPS, lipopolysaccharide; S100A9, S100 calcium-binding protein A9; MyD88, myeloid differentiation primary TAK-binding proteins 1; IKK, IκB kinase; NEMO, NF-κB essential modulator; Mal, MyD8response 88; RIP2, serine–threonine protein 2; SYK, spleen tyrosine kinase; IRAK4, IL-1R-related kinase 4; TRAF6, TNF receptor-associated factor 6; TAK1, TGF-β-activated kinase 1; TAB1, 8-adapter-like; TRIF, TIR domain-containing adaptor protein-inducing interferon β; TRAM, TRIF-related adaptor molecule; NOD, nucleotide oligomerization domain; cGAS, cyclic GMP–AMP synthase; cGAMP, 2′3′ cyclic GMP–AMP; STING, stimulator of interferon genes; TBK1, TANK-binding kinase 1; RIG-I, retinoic acid-inducible gene-I; MDA5, melanoma differentiation-associated gene 5; MAVS, mitochondrial antiviral signaling protein; TANK, TRAF family member-associated NFKB activator; SINTBAD, similar to NAP1 TBK1 adaptor; IRF3, interferon regulatory factor 3; CARD9, caspase activation and recruitment domain 9; BCL10, B-cell lymphoma/leukemia 10; MALT1, mucosa-associated lymphoid tissue lymphoma translocation protein 1; JNK, c-Jun N-terminal kinase; MAPK, mitogen-activated protein kinase; AP-1, activator protein 1; NLRP3, NLR family pyrin domain containing 3; ASC, apoptosis-associated speck-like protein containing CARD; Arg1, Arginase1; iNOS, inducible nitric oxide synthase; COX2, cyclooxygenase-2; TNF, tumor necrosis factor; PD-L1, programmed death-ligand 1; CXCR4, C-X-C chemokine receptor type 4; dsRNA, double-strand RNA; ssRNA, single-strand RNA; IFN-β, interferon-β; TNF-α, tumor necrosis factor-α; CXCL9, C-X-C motif chemokine ligand 9; CCL4, C-C motif chemokine ligand 4.

Despite the large number of PRRs discovered as well as their ligands, there are only a few types of signal transduction pathways that are shared by them, and all pathways are cross-talking. As we gained deeper insight into the role of MDSCs in cancer, these pathways were found to be involved in MDSC polarization and immunosuppressive function (**Figure 2**).

### NF-κB signaling

Nuclear factor kappa-light-chain-enhancer of activated B cells (NF-κB) plays a key role in the process of cellular inflammation and immune response. NF-κB is a heterodimer composed of two molecules, p50 and p65, and it is usually inactive by binding to the inhibitory protein IκB under normal conditions. Toll-like receptors are considered universally conserved activators of NF-κB signaling in MDSCs (93).

After TLRs bind to corresponding PAMPs and DAMPs, the TIR domains conduct signals by myeloid differentiation primary response 88 (MyD88)-dependent and MyD88-independent pathways. MyD88 binds to the TIR domain of TLRs, recruits IL-1R-related kinase 4 (IRAK4) and activates IRAK1 and IRAK2. Ubiquitin ligase TNF receptor-associated factor 6 (TRAF6) is recruited to form a complex with TGF-β-activated kinase 1 (TAK1) and two TAK-binding proteins (TAB1 and TAB4). TRAF6 is then degraded. The TAK1–TAB1–TAB4 complex then phosphorylates the IκB kinase (IKK) complex, causing its own degradation by ubiquitination. Afterward, NF-κB is released and translocated into the nucleus, thereby regulating the transcription of inflammatory genes, including TNF, IL-6, IL-1, and other chemokines (8). Recently, it was reported that murine fibrosarcoma-derived PGE2 induced the accumulation of p50 in the nucleus, which led to strengthened binding of STAT1 to selected IFNγ-dependent genes in M-MDSCs, resulting in higher NOS2 expression. This process modulated M-MDSCs into an NO-mediated immunosuppression phenotype and reduced TNFα expression that can support tumors in return (94). Furthermore, the cellular FADD-like IL-1β-converting enzyme-inhibitory protein expression in M-MDSCs of PDAC patients was reported to be increased significantly, which played a major role in chemotherapy resistance, including commonly applied 5-fluorouracil (5-FU) and Gem, in part through the activation of the canonical NF-κB signaling pathway (20).

The immunosuppression function directed by NF-κB signaling has been well described in anti-inflammatory processes after long-term TLR activation. Sustained activation of TLRs causes persistent production of proinflammatory cytokines, such as TNF or IL-6, leading to tissue damage (95, 96). Similarly, PRR activation in MDSCs also plays an important role in the cancer process. Tumor cells can generate abnormal surface components that activate PRRs on MDSCs. Melanoma cells change the glycocalyx structure on their heparan sulfate proteoglycan, which can bind to TLR2 on MDSCs and facilitate the recruitment of MDSCs *via* the TLR2/MyD88/IL‐6/STAT3 pathway, leading to the inhibition of NK cell recruitment and cytotoxicity and ultimately tumor progression and metastasis (97). Melanoma can also release heat shock protein 86, acting as a DAMP, which is recognized by TLR4 on MDSCs. Both adaptors (MyD88 and TRIF) accumulate to activate NF-κB, with a pronounced expression of IL1β, IL6, IL10, TNFα, COX-2, and PD-L1, leading to a strong immunosuppressive capacity toward T lymphocytes (98). S100A9 was found in the CRC microenvironment to activate the MDSC immune suppressive program through TLR4-dependent NF-κB signaling pathways, resulting in upregulated Arg1, iNOS, and IL-10 production (55). Tumors exploit extracellular vesicles (EVs) to deliver long-distance information that can regulate the host immune system. Renal cancer-derived exosomes encapsulate HSP70 to recognize TLR2 and promote MDSC expansion and production of iNOS, ARG1, and ROS through the MyD88/TRAF6/P38/AP-1 pathway (99). Exosomes derived from prostate cancer cells can upregulate CXC motif receptor 4 (CXCR4) *via* the TLR2/NF-κB signaling pathway, eventually promoting the migration of MDSCs into the tumor microenvironment in a CXCR4–CXCL12 axis-dependent manner (100). Conventional monocytes readily take up palmitoylated proteins from acute myeloid leukemia-derived EVs, activating the TLR2/NF-κB signaling pathway, and subsequently undergo MDSC differentiation, expressing a typical HLA-DR^low^ phenotype, releasing IDO-1, and gaining T cell suppression ability (101). Nevertheless, some TLRs can also be activated to abstain from MDSC immunosuppressive functions. In contrast to glucans, nCKAP-2, a branched arabinan-type polysaccharide purified from *Curcuma kwangsiensis*, was discovered to exert an anti-MDSC effect by inducing the apoptosis of MDSCs through the TLR4-mediated NF-κB signaling pathway (102). Mifamurtide, a prodrug that can release free muramyl dipeptide and initiate NOD2-NF-κB signaling in patients with cancer, has been successfully used to treat osteosarcoma with markedly reduced MDSC accumulation (103).

### MAPK signaling

Mitogen-activated protein kinase (MAPK) signaling is an evolutionarily conserved pathway linking extracellular signals to fundamental cellular processes such as growth, proliferation, differentiation, migration, and apoptosis. In the MyD88-dependent pathway of TLRs, IRAK-1 is activated and interacts with TRAF6 and then activates the IKK complex. In this step, IRAK-1 can also cause the activation of MAPKs, including three subfamilies: c-Jun N-terminal kinase (JNK), p38, and MAPK. When NLRs are activated because they sense bacterial components, downstream CARD9 can be recruited, thereby activating p38, JNK, and finally the MAPK pathway to promote the release of proinflammatory factors.

MAPK is reported to modulate MDSC plasticity. LPS could convert the potentially suppressive Gr1^+^CD115^+^ monocytes toward an inflammation stimulatory monocyte *via* the p38/MAPK signaling pathway, reducing its ability to convert conventional CD4^+^ T cells into CD4^+^CD25^+^Foxp3^+^ Tregs (104). The ERK/MAPK pathway inhibition was related to suppressed tumor growth and promoted MDSC apoptosis (105). Immune checkpoint protein V-domain immunoglobulin suppressor of T cell activation dampened the TLR-mediated activation of both MAPK/AP-1 and IKK/NF-κB signaling cascades and restored T cell IFN-γ production in mice bearing melanoma and increased the cytokines IL12p40, IL12p70, IL23p19, IL6, IFNβ, and TNFα as well as the chemokines CCL4, CCL5, CXCL9, and CXCL10 inside the tumor tissue (106). Nevertheless, the complement system can sense DAMPs and apoptotic cells and trigger a sterile inflammatory response. Although complement activation on the tumor endothelium can reverse the endothelial barrier and fuel T cell entrance into the tumor microenvironment (107), C5a orchestrates MDSC infiltration and the immune suppression program in tumors by regulating the production of ROS and RNS (108). Accordingly, C3 can be produced by hepatic carcinoma cells to activate MDSCs to produce IL10 through p38/MAPK signaling (109).

### TBK1–IRF-3 signaling

The major adaptor protein in the MyD88-independent pathway is TRIF, which mainly induces type I IFN expression. After binding to its cognate receptor, TRIF and TRAF3 activate signaling, leading to the recruitment of IKKϵ/TANK-binding kinase 1 (TBK1), which can phosphorylate IRF3 and activate type I IFN gene transcription. This cascade of events regulates innate anti-viral immune responses. RLRs such as RIG-I and MDA5 can detect viral RNAs. Then, MDA5 and RIG-I interact with their common adaptor mitochondrial antiviral signaling protein to dimerize and bind to TRAF3. In turn, TRAF3 recruits the adaptor proteins TANK, NAP1, and SINTBAD, which induce the phosphorylation of IRF-3 (8). IFN plays a central role in antiviral immunity and was recently considered to directly affect MDSC function (110). Importantly, IFN-I signaling in PMN-MDSCs was a prerequisite for their immunosuppressive effects, and the downregulation of IFN-I signaling in PMN-MDSCs led to the activation of the PI3K-Akt/mTOR pathway, which led PMN-MDSCs to obtain their immunosuppressive traits in peripheral blood (111).

### Inflammasome signaling

Inflammasomes are cytosolic macromolecular complexes assembled by multiple PRRs in the cytoplasm. There are five types of inflammasomes, including the NLRP1 inflammasome, NLRP3 inflammasome, NLRC4 inflammasome, IPAF inflammasome, and AIM2 inflammasome. Inflammasomes are usually composed of apoptosis-associated speck-like protein containing a CARD (ASC), caspase protease, and a protein of the NLR family (*e*.*g*., NLRP3) or HIN-200 family protein (*e*.*g*., AIM2). Taking NLR inflammasome formation as an example, the dimerization of NLRs under the action of intracellular PAMPs or DAMPs makes pyrin domains (PYDs) polymerize and then activate the ASC complex, which activates the effector complex composed of CARD and caspase-1. Finally, NLRs (LRR + NACHT + PYD), ASC (PYD + CARD), and the effector complex (CARD + caspase-1) together constitute the inflammasome complex. After formation, inflammasomes can activate caspase-1. Activated caspase-1 splices proIL-1β/proIL-18 into the corresponding mature cytokines. NLRP3 is the most studied inflammasome sensor driving IL-1β-mediated conditions from sterile inflammation to rare hereditary syndromes (76).

MDSCs exhibit an increased activation of NLRP3 during tumor development. Ablation of the NLRP3/pro-IL-1β inflammasome rewires MDSC function, promotes melanoma (112) and breast cancer (113) regression, and causes cisplatin resistance (114). NLRP3 inhibition and anti-PD-1 treatment significantly increased the antitumor efficacy of monotherapy by limiting MDSC-mediated T cell suppression and melanoma tumor progression (76). Gemcitabine and 5-fluorouracil were reported to be able to activate the NLRP3 inflammasome in MDSCs, leading to the production of IL-1β, and IL-1β induced the secretion of IL-17 by CD4^+^ T cells, which blunted the chemotherapy efficacy (74).

However, diverse functions of the NLRP3 inflammasome in MDSCs have also been reported. The expression of the scavenger receptor macrophage receptor with collagenous structure (MARCO) on MDSCs correlates with poor prognosis and a “cold tumor” phenotype in PDAC patients. Anti-human MARCO (anti-hMARCO) antibody activated the NLRP3 inflammasome, resulting in IL-18 production, and could help T cells and NK cells produce IFNγ (115). Nevertheless, melanoma expresses high NLRP3-derived IL-1β expression, which specifically induces pSTAT3 and amplifies IL-6 secretion through the IL-1β/IL-6/STAT3 axis *in vivo*, leading to a further expansion of MDSCs (77). A previous preclinical report suggested that the limitation of 5-FU anticancer efficacy is due to IL-1β secretion by MDSCs, 5-FU-mediated NLRP3 activation, and subsequent caspase-1 activity in MDSCs driving the expression of IL-1β. The application of docosahexaenoic acid improved 5-FU efficacy by inhibiting IL-1β secretion and caspase-1 activity in an MDSC cell line (MSC-2) (116). Mifamurtide, except from binding to NOD2, also activates NLRP3 and promotes the activation of IL-1β in macrophages and monocytes. Application of mifamurtide significantly improves event-free survival and overall survival in osteosarcoma patients (117).

### The STING pathway

The detection of cytosolic DNA is contributed, in a large part, by the cyclic GMP–AMP synthase (cGAS)–stimulator of interferon genes (STING) pathway, which has emerged as a critical mechanism for coupling the sensing of cellular perturbation to the powerful innate immune responses. In this pathway, after sensing dsDNA, cGAS allosterically activates its catalytic activity and leads to the production of 2′3′ cyclic GMP–AMP (cGAMP), a potent agonist of the endoplasmic reticulum (ER) membrane protein STING. Then, STING translocates from the ER to the Golgi, where STING can bind to TBK1 and then recruit and phosphorylate IRF3 and subsequently activate the production of type I IFNs.

The protumor *vs*. antitumor roles of STING have been argued. In B16 melanoma tumor-bearing mice, an intravenous administration of the STING agonist cGAMP decreased the number of GR1^+^ and especially Ly6G^+^ PMN-MDSCs in the spleen and tumor while suppressing the production of ROS and NO from MDSCs and abolishing their suppressive function (118). The application of cyclic diguanylate (c-di-GMP), a ligand for STING, significantly increased the production of IL-12 by MDSCs, reversing its suppressive function, in correlation with an improved vaccination response against metastatic breast cancer (119). The intrinsic activation of the unfolded protein response mediator PKR-like ER kinase (PERK) in MDSCs suppressed the STING pathway in cancer by decreasing the STING-driven production of type I IFN (120). Moreover, independent of IFN production, STING overexpression in MDSCs upregulated the suppressor of cytokine signaling 1 (SOCS1), a potent inhibitor of Janus kinase (JAK) 1/2 in the JAK/STAT signaling pathway (121), preventing STAT3 phosphorylation (122, 123) and inhibiting the production and release of IL-6 and GM-CSF, two known drivers of MDSC expansion in nasopharyngeal carcinoma (7).

## TLRs as potential targets for MDSCs in cancer

PRRs widely exist in tumors and are not only related to the growth and proliferation of tumors themselves but are also closely related to the formation of the tumor microenvironment (124). Therefore, how to utilize and target these receptors has aroused extensive interest from researchers. Among PRRs, TLRs are the most widely studied and modulated. Tumor cells release DAMPs actively or when they die, such as HMGB1, HSP, and S100 (44). MDSCs capture these molecules to respond to these proteins through the TLR pathway to increase activity, expand proliferation, or increase penetration. A variety of drugs targeting TLRs have been developed and even used in the treatment of clinical tumor patients or combined with immune checkpoint inhibitors (ICIs). The major treatment strategies targeting PRRs are to use ligand analogs to activate them, antagonists to inhibit their activation, or antibodies and small molecules to inhibit the related signaling pathways (125). Today, most targeted drugs are mostly in the preclinical development stage. Since this future therapeutic approach will be developed not only to directly target tumor cells but also to modify the immune composition of the TME, targeting different pro-tumor immune cells *via* TLRs has also attracted attention, whether combined with ICIs (**Table 1**) or with other therapies (**Table 2**). Unfortunately, only a few clinical trials nowadays aim to evaluate the therapeutic impact of TLR targeting in reprogramming MDSC cell functionality and activity (**Tables 1**, **2**).

**Table 1:** Summary of clinical trials targeting Toll-like receptor (TLRs) in combination with immune checkpoint inhibitors.

TLR targets	Drug name	Combining targets	Drug name	Indications	Phase	Last report status	NCT number	Myeloid-derived suppressor cell as outcome measure
TLR 3	Poly-ICLC (agonist)	Anti-PD1	Pembrolizumab	Colon cancer	I/II	Recruiting	NCT02834052	No
	Poly-ICLC (agonist)	Anti-PD-L1 + anti-CTLA-4	Durvalumab, tremelimumab	Advanced cancers	I/II	Completed	NCT02643303	No
	Rintatolimod (agonist)	Anti-PD1 + chemotherapy	Pembrolizumab, cisplatin	Ovarian cancer	I/II	Recruiting	NCT03734692	No
	Poly-ICLC (agonist)	Anti-PD1 + ultrasound ablation		Advanced solid tumors	I	Recruiting	NCT04116320	No
	BO-112 (agonist)	Anti-PD1	Pembrolizumab	Melanoma	II	Active, not recruiting	NCT04570332	No
TLR 4	GSK1795091 (agonist)	Anti-PD1	Pembrolizumab	Advanced solid tumors	I	Completed	NCT03447314	No
TLR 7	SHR2150 (agonist)	Anti-PD-1 or anti-CD47 + chemotherapy		Solid tumors	I/II	Unknown	NCT04588324	No
	DSP-0509 (agonist)	Anti-PD1	Pembrolizumab	Advanced solid tumors	I/II	Recruiting	NCT03416335	No
	BNT411 (agonist)	Anti-PD-L1 + chemotherapy	Atezolizumab, carboplatin, and etoposide	Solid tumors	I/Iia	Recruiting	NCT04101357	No
TLR 8	Motolimod (agonist)	Anti-PD1	Nivolumab	Head and neck cancer	I	Completed	NCT03906526	No
	Motolimod (agonist)	Anti-PD-L1	Durvalumab	Ovarian cancer	I/II	Completed	NCT02431559	No
	SBT6050 (agonist)	Anti-PD1	Pembrolizumab and cemiplimab	Advanced solid tumors expressing HER2	I/Ib	Active, not recruiting	NCT04460456	No
TLR 7/8	TransCon TLR7/8 (agonist)	Anti-PD1	Pembrolizumab	Advanced or metastatic solid tumors	I/II	Recruiting	NCT04799054	No
	BDB018 (agonist)	Anti-PD1	Pembrolizumab	Advanced solid tumors	I	Recruiting	NCT04840394	No
	BDC-1001 (agonist)	Anti-PD1	Nivolumab	HER2 positive solid tumors	I/II	Recruiting	NCT04278144	No
	BDB001 (agonist)	Anti-PD-L1	Atezolizumab	Advanced solid tumors	I	Active, not recruiting	NCT04196530	No
	BDB001 (agonist)	Anti-PD1	Pembrolizumab	Advanced solid tumors	I	Active, not recruiting	NCT03486301	No
TLR 9	MGN1703 (agonist)	Anti-CTLA-4	Ipilimumab	Advanced solid malignancies and melanoma	I	Active, not recruiting	NCT02668770	No
	Tilsotolimod (agonist)	Anti-PD1 + anti-CTLA-4	Ipilimumab and nivolumab	Advanced cancers	I	Active, not recruiting	NCT04270864	No
	SD-101 (agonist)	Anti-CTLA-4 + radiation	Ipilimumab	B-cell lymphoma	I/II	Completed	NCT02254772	No
	Vidutolimod (agonist)	Anti-PD1	Nivolumab	Prostate cancer	II	Not yet recruiting	NCT05445609	No
	Vidutolimod (agonist)	Anti-PD1	Pembrolizumab	Melanoma	II	Recruiting	NCT04708418	No
	SD-101 (agonist)	Anti-PD1	Pembrolizumab	Pancreatic cancer	I	Recruiting	NCT05607953	No
	DV281 (agonist)	Anti-PD-1		Non-small cell lung cancer	I	Completed	NCT03326752	No
	SD-101 (agonist)	Anti-PD1 + radiation	Pembrolizumab	Prostate cancer	II	Active, not recruiting	NCT03007732	No
	Vidutolimod (agonist)	Anti-PD1	Nivolumab	Melanoma	II	Recruiting	NCT04401995	No
	Vidutolimod (agonist)	Anti-4-1BB/CD137 + anti-PD-L1	Utomilumab and avelumab	Advanced cancer	Ib/II	Active, not recruiting	NCT02554812	No
	SD-101 (agonist)	Anti-PD1 + anti-CTLA-4	Nivolumab and Ipilimumab	Melanoma	I	Recruiting	NCT04935229	No
	Vidutolimod (agonist)	Anti-PD1 + anti-CTLA-4 + radiation	Nivolumab, ipilimumab	Colorectal cancer with liver metastases	I	Active, not recruiting	NCT03507699	No
	**Vidutolimod (agonist)**	**Anti-PD1**	**Nivolumab**	**Melanoma**	**II**	**Active, not recruiting**	**NCT03618641**	**Yes**
	SD-101 (agonist)	Anti-PD1	Nivolumab and radiation therapy	Pancreatic Cancer	I	Active, not recruiting	NCT04050085	No
	SD-101 (agonist)	Anti-PD1 + anti-CTLA-4	Pembrolizumab, nivolumab and ipilimumab	Primary liver tumors	I/II	Recruiting	NCT05220722	No
	Vidutolimod (agonist)	Anti-OX40 antibody	INCAGN01949	Pancreatic cancer	Ib/II	Recruiting	NCT04387071	No
	SD-101 (agonist)	Anti-OX40 antibody	BMS-986178	Advanced solid tumors	I	Active, not recruiting	NCT03831295	No
	SD-101 (agonist)	Anti-OX40 antibody + radiation	BMS-986178	B-Cell non-Hodgkin lymphomas	I	Active, not recruiting	NCT03410901	No

The bold entries mean that they are the clinical trials taking MDSCs as outcome measure.

**Table 2:** Summary of clinical trials targeting Toll-like receptor (TLRs) as monotherapy or in combination with other treatments.

TLR targets	Drug name	Combining therapy	Drug name	Indications	Phase	Last report status	NCT number	Myeloid-derived suppressor cell as outcome measure
TLR 1/2	XS15 (agonist)	Vaccine	Personalized multi-peptide vaccine	Advanced solid and hematological malignancies	Not applicable	Available	NCT05014607	No
	XS15 (agonist)	Vaccine	Multi-peptide vaccine	Leukemia	I	Recruiting	NCT04688385	No
TLR 3	Poly-ICLC (agonist)	Vaccine	CDX-1140,6MHP and NeoAg-mBRAF	Melanoma	I/II	Recruiting	NCT04364230	No
	Poly-ICLC (agonist)	Vaccine	NY-ESO-1 protein vaccination	Melanoma	I/II	Completed	NCT01079741	No
	Poly-ICLC (agonist)	Cell infusion	GM-CSF+ CAR-T or TCR-T	Gliomas	I	Unknown	NCT03392545	No
TLR 2/4	CX-01 (inhibitor)	Chemotherapy	Azacitidine	Leukemia	I	Completed	NCT02995655	No
TLR 4	GLA-SE (agonist)	Radiotherapy		Sarcoma	I	Completed	NCT02180698	No
	GLA-SE (agonist)	Vaccine	MART-1a peptide or antigen Vaccines	Melanoma	I	Completed	NCT02320305	No
TLR 5	Entolimod (agonist)	Monotherapy		Colorectal cancer	II	Unknown	NCT02715882	No
	Entolimod (agonist)	Monotherapy		Advanced solid tumors	I	Completed	NCT01527136	No
TLR 7	852A (agonist)	Monotherapy		Breast, ovarian, endometrial and cervical cancers	II	Completed	NCT00319748	No
	Imiquimod (agonist)	Monotherapy		Oral cancer	I	Recruiting	NCT04883645	No
	Imiquimod (agonist)	Chemotherapy	Topical fluorouracil	Cervical intraepithelial neoplasia	I	Active, not recruiting	NCT03196180	No
	Imiquimod (agonist)	Chemotherapy + radiotherapy	Cyclophosphamide	Breast cancer	II	Completed	NCT01421017	No
	Imiquimod (agonist)	Laser therapy		Melanoma	I	Completed	NCT00453050	No
	NJH395 (agonist)	Anti-HER2 antibody		Non-breast HER2+ advanced malignancies	I	Completed	NCT03696771	No
	TQ-A3334 (agonist)	Small molecule kinase inhibitor	Anlotinib	Non-small cell lung cancer	I/II	Unknown	NCT04273815	No
TLR 8	Motolimod (agonist)	Chemotherapy	Doxorubicin or paclitaxel	Ovarian epithelial, fallopian tube, or peritoneal cavity cancer	I	Completed	NCT01294293	No
	**Motolimod (agonist)**	**Chemotherapy**	**Cyclophosphamide**	**Metastatic, persistent, recurrent, or progressive solid tumors**	**I**	**Terminated**	**NCT02650635**	**Yes**
	Motolimod (agonist)	Small molecule kinase inhibitor	Cetuximab	Squamous cell cancer of head and neck	I	Completed	NCT01334177	No
	Motolimod (agonist)	Small molecule kinase inhibitor + chemotherapy	Cisplatin or carboplatin + 5-fluorouracil + cetuximab	Squamous cell carcinoma of the head and neck	II	Completed	NCT01836029	No
TLR 7/8	BDB001 (agonist)	Monotherapy		PD-(L)1 refractory solid tumors	II	Active, not recruiting	NCT04819373	No
	Resiquimod (agonist)	Vaccine	Gp100(g209-2M) and MAGE-3 vaccines,	Melanoma	II	Completed	NCT00960752	No
	Resiquimod (agonist)	Vaccine	NY-ESO-1 protein vaccination	Tumors express NY-ESO-1	I	Completed	NCT00821652	No
TLR 3 or TLR 7/8	Poly-ICLC (agonist) or resiquimod (agonist)	Vaccine	Peptide vaccine (LPV7) + tetanus peptide	Melanoma	I/II	Unknown	NCT02126579	No
	Poly-ICLC (agonist) or resiquimod (agonist)	Vaccine	Tumor-lysate pulsed DC vaccination	Brain tumors	II	Active, not recruiting	NCT01204684	No
TLR 9	Tilsotolimod (agonist)	Monotherapy		Melanoma	II	Recruiting	NCT04126876	No
	CpG-STAT3 siRNA CAS3/SS3 (agonist)	Radiotherapy		B-Cell non-Hodgkin lymphoma	I	Recruiting	NCT04995536	No
	CPG-7909 (agonist)	Radiotherapy		Lymphomas	I/II	Completed	NCT00185965	No
	CpG-7909 (agonist)	Vaccine	Tumor specific epitope peptides vaccine	Esophageal cancer	I/II	Unknown	NCT00669292	No
	DUK-CPG-001 (agonist)	Cell infusion	NK cell-enriched donor lymphocyte infusions	Myeloid malignancies or lymphoid malignancies	II	Recruiting	NCT02452697	No
	SD-101 (agonist)	Small molecule kinase inhibitor	Ibrutinib	Follicular lymphoma	Ib/II	Active, not recruiting	NCT02927964	No
	EMD 1201081 (agonist)	Small molecule kinase inhibitor	Cetuximab	Head and neck squamous cell carcinoma	II	Completed	NCT01040832	No
TLR 7,8, and 9	IMO-8400 (antagonist)	Monotherapy		Diffuse large B cell lymphoma	I/II	Completed	NCT02252146	No

The bold entries mean that they are the clinical trials taking MDSCs as outcome measure.

### TLR1/2

Studies on TLR1/2 agonists in MDSCs are controversial. Pam3CSK4, an agonist of TLR1/2, can promote the differentiation of M-MDSCs in the blood of patients with colon, prostate, pancreas, or liver cancer into M2-type macrophages with highly immunosuppressive functions (34). In a melanoma study, Pam3CSK4 upregulated the expression of PD-L1 on immature myeloid cells (IMCs) (98). Both studies suggest that the activation of TLR1/2 on MDSCs promotes the formation of an immunosuppressive microenvironment. However, in another mouse model of HCC, Pam3CSK4 helps M-MDSCs polarize toward mature macrophages and DCs and exert antitumor functions to prevent tumor progression (126). In lung cancer, Deng et al. found that applying agonists of TLR1 and TLR2 using bacterial lipoprotein not only inhibited tumor growth but also reduced the number of M-MDSCs in mouse models (48). A more interesting study on colon cancer found that an important DAMP molecule released by dying tumor cells, translational control tumor protein (TCTP), activates TLR2 on M-MDSCs and promotes the secretion of CXCL1/2, recruiting PMN-MDSCs into the TME. They abolished TCTP using 55F3, a monoclonal antibody, and found reduced CXCL1/2 concentrations as well as reduced PMN-MDSC infiltration (46).

### TLR3

Double-stranded RNA (dsRNA), released by viruses or damaged cells, is the common ligand for TLR3. The TLR3 agonist poly(I:C)/PIC, an artificial dsRNA analog that has significant potential to enhance the immune system, has been widely used in cancer therapy (127, 128). One recent study on lymphoma using irreversible electroporation combined with anti-PD1 and TLR3/9 agonists indicated a sound treatment response by reversing the immunosuppressive TME, including the reduction in MDSCs (65). PIC combined with low-dose cisplatin showed better drug tolerance in treating oral squamous cell carcinoma and enhanced antitumor effects (129). Reovirus has been actively studied as an oncolytic virus. Its antitumor immune effect is also considered to be closely related to TLR. When reovirus was internalized by MDSCs, the expression of TLR3 on MDSCs was significantly upregulated, accompanied by a decrease in immunosuppressive ability (130).

### TLR4

HMGB1, as a DAMP released by cancer cells, can efficiently activate TLR4. Blocking HMGB1 led to remodeling of the TME, including a marked reduction in MDSCs. Although blocking HMGB1 alone did not improve survival, it enhanced the response to anti-PD1 therapy (131). Another study showed that, in lung cancer, tumor-dependent complement 5a increases the expression of the HMGB1 receptors TLR4 and RAGE (advanced glycation end products) on PMN-MDSCs, promoting their neutrophil extracellular trap (NET) generation. NETs encapsulate and shelter circulating tumor cells from attack by immune cells to support tumor metastasis in lung cancer. When the release of HMGB1 was inhibited using glycyrrhizin, NET formation was significantly reduced (132). The coculture of melanoma-derived extracellular vesicles with IMCs transformed them into immunosuppressive MDSCs with upregulated PD-L1 expression *in vitro*. However, when melanoma-derived EVs were added to TLR4-knockout IMCs, the upregulation of PD-L1 was blocked (98).

### TLR5

Flagellin, a structural protein of the flagellum of bacteria, is the only known TLR5 ligand. An ongoing phase I study (NCT01527136) has shown that flagellin is well tolerated and relatively safe. Engated T cells with flagellin-secreting ability reshaped the TME by delivering the agonist locally at the tumor site and demonstrated good antitumor effects with reduced infiltration of MDSCs (133).

### TLR7/8

TLR7/8 are the other hot targets among TLRs. R848, a classical TLR7/8 agonist, showed a profound influence on MDSCs. MDSCs isolated from the peripheral blood of breast cancer patients and stimulated with R848 showed a significantly reduced viability, while antigen-presenting cells such as dendritic cells and macrophages increased significantly (134). R848 injection in colon tumors induced a significant reduction in MDSCs in mice (69). Moonkyu Lee et al. also cultured MDSCs with R848 conditioned medium *in vitro* and confirmed their reduced expansion (135). R848 can even improve the drug resistance of oxaliplatin during the treatment of colon cancer, which promotes the differentiation of MDSCs into M1 macrophages, modifying the immunosuppressive TME and enhancing the antitumor effect of oxaliplatin (136). Other TLR7/8 agonist-related clinical trials are underway, such as the combination of cyclophosphamide and the TLR8 agonist VTX-2337 in the treatment of solid tumors (NCT02650635). A phase Ib clinical study combining neoadjuvant cetuximab and the TLR8 agonist R848 in head and neck cancer showed hampered MDSC suppressive signals in an NF-kB-mediated manner (137).

### TLR9

The role of TLR9 in MDSCs is also controversial and may be relevant to cancer types. Synthetic unmethylated CpG oligodeoxynucleotide (ODN), a commonly utilized TLR9 receptor agonist, did not achieve the expected results in clinical trials when used alone; however, great results characterized by an increase in the antitumor T cell-mediated immune response have been achieved in a plethora of clinical trials in which CpG-ODN has been synergistically codelivered with other therapeutic approaches (40, 138, 139). Studies have shown that CpG can effectively block the inhibitory activity of MDSCs (67). In liver cancer, local application of the TLR agonist ODN2395 in tumors induced the differentiation of MDSCs into mature macrophages (140). When using CpG-siRNA conjugates that specifically silence STAT3 in TLR9-positive myeloid cells in mouse prostate cancer cells, the downstream STAT3 pathway activation blockage decreased the expression and activity of Arg-1, thus weakening the immunosuppression ability of MDSCs (141). In pancreatic cancer, CpG application leads to the recruitment of regulatory T cells and the proliferation of MDSCs by binding to TLR9 (124). Furthermore, treatment with ODN2395 and SD101 induced MDSC apoptosis and increased the M1/M2 macrophage ratio in an NFκB-dependent manner (140).

There are many PRRs worth investigating, such as the NLRP family, RLRs, and CLRs. However, even for the TLR family, what role it plays in MDSCs, when it needs to be stimulated, or when it needs to be blocked is still an open question. Regarding the widespread use of TLR-related drugs and their strong impact on the tumor immune microenvironment, in-depth related research is worthwhile.

## Conclusions and perspectives

**Figure 3:**
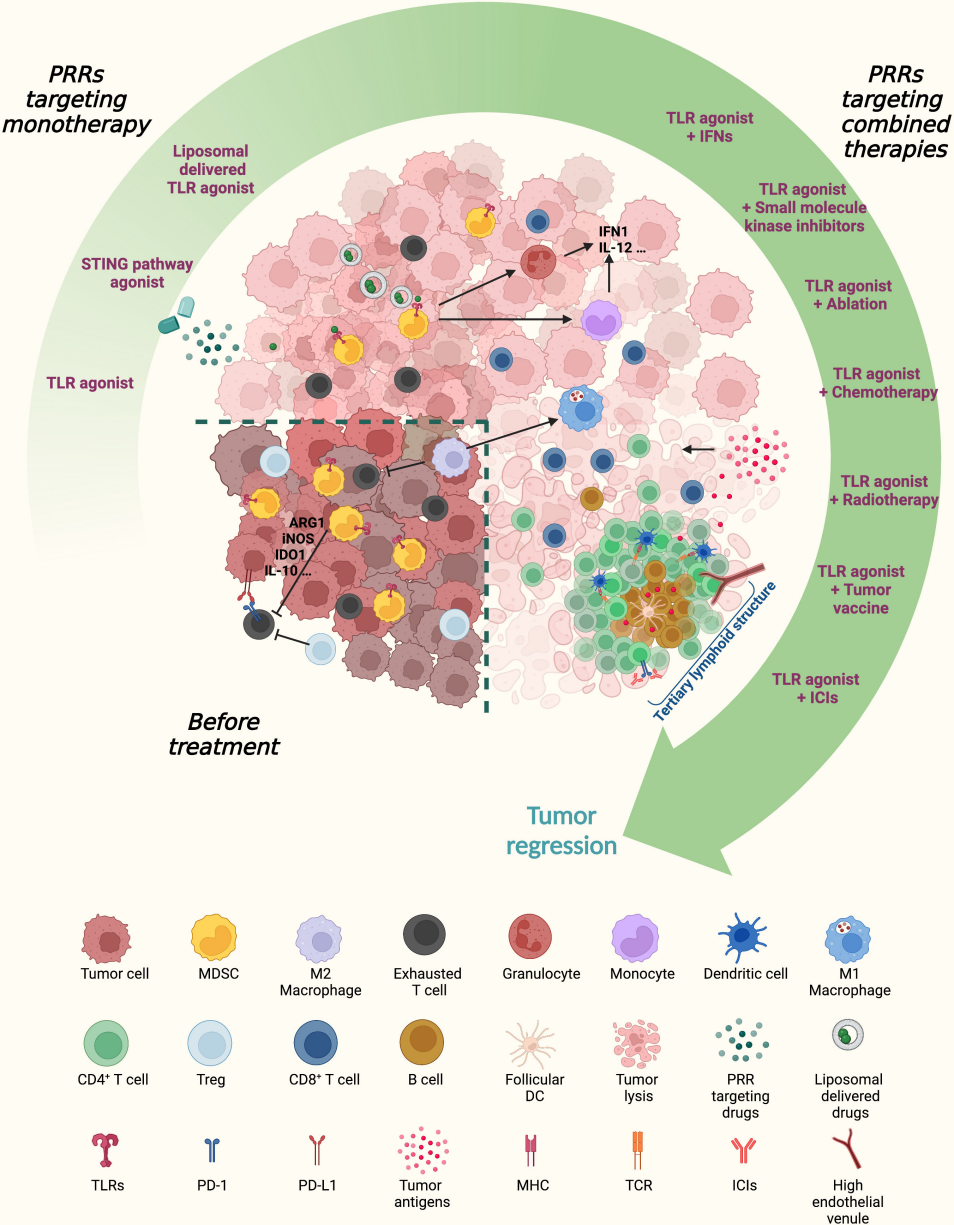
Next-generation PRR-based therapeutic approaches for cancer patients. PRR-targeting agonists, especially when combined with anti-inflammatory and/or immunostimulatory agents, can switch an immunosuppressive tumor microenvironment (TME) hostile for T cell trafficking and fitness toward an immunogenic milieu. PRR agonists promote MDSC maturation into inflammatory monocytes and granulocytes and increase the M1/M2 macrophage ratio. Combining PRR-targeting therapies with classic immunotherapy might support the establishment of TLSs containing a CD3^+^ T cell zone with mature dendritic cells (DCs) and a germinal center with B cells and follicular DCs. The coordinated actions of tumor antigen presentation through DCs and cytotoxic effector T cells and B cells enable the *in situ* priming and sustainment of antitumor adaptive immunity. MDSCs, myeloid-derived suppressor cells; DCs, dendritic cells; Treg, regulatory T cell; PRRs, pattern recognition receptors; TLR, Toll-like receptor; STING, stimulator of interferon genes; IFN, interferon; ICIs, immune checkpoint inhibitors; Arg1, arginase 1; iNOS, inducible nitric oxide synthase; IDO1, indoleamine 2,3-dioxygenase 1; PD1, programmed cell death protein 1; PD-L1, programmed death-ligand 1; TLS, tertiary lymphoid structure; MHC, major histocompatibility complex; TCR, T cell receptor.

The wide range of PRRs affords a potentially unprecedented opportunity to arm the cancer immunotherapy arsenal. Their ability not only to link innate and adaptive immunity but also to shape numerous aspects of the immune response in the tumor microenvironment, such as the modulation of cancer cells themselves, makes PRR-targeting drugs continuously growing. Most studies on the PRR family have used TLR agonists in clinical trials. Combining PRR agonists with conventional cancer therapies showed enhanced antitumor efficacy (**Figure 3**). Motolimod in combination with chemotherapy produced clinical benefits in ovarian cancer and HPV-positive head and neck squamous cell carcinoma patients (142, 143) (**Table 2**). Moreover, TLR9 agonists combined with targeted therapy, such as the small molecule kinase inhibitor cetuximab, showed good tolerability, although there was no incremental clinical efficacy in SCCHN patients (144), suggesting that more studies are required to achieve final conclusions. Ionizing radiation can activate the cGAS/STING pathway to trigger tumor antigen uptake and cross-presentation, inducing antitumor T cell immunity, and several clinical trials are currently testing the efficacy of radiation therapy in combination with TLR agonists (145) (**Table 2**). Similarly, tumor vaccination together with the TLR4 agonist GLA (glucopyranosyl) proved to have good tolerability in melanoma patients, and the clinical results will soon be disclosed (146). Other combinations (IFN-γ, Poly I:C plus STING agonists) are still in the preclinical testing stage and have shown promising results (147).

At present, the greatest enthusiasm is for the association of TLR agonists with immune checkpoint-targeted inhibitors (such as anti-CTLA-4 or anti-PD-L1 antibodies), as what emerged from the ongoing study evaluating the efficacy of the intratumoral TLR9 agonist tilsotolimod (IMO-2125) in combination with ICIs such as ipilimumab and nivolumab in different solid tumors (ILLUMINATE-206). TLR9 agonists in combination with nivolumab have already shown prolonged disease control in advanced NSCLC patients (NCT03326752). The TLR4 agonist GSK1795091 plus immunotherapy [GSK3174998 (anti‐OX40 monoclonal antibody), GSK3359609 (anti‐ICOS monoclonal antibody), or pembrolizumab] showed good safety performance in patients with solid tumors (148) (**Table 2** and **Figure 3**).

Moreover, the continuous understanding of the immune regulation mechanism of PRRs along with the burgeoning of nanotechnology has improved targeting and therapeutic effects in fighting tumor immune escape (**Figure 3**). A tumor-targeting liposomal formulation (PCL8-U75) that arouses cytotoxic effects, particularly depleting MDSCs and tumor-associated macrophages in the TME, has been investigated in combination with immune-activating therapies such as the TLR7 agonist R848, which showed a potent antitumoral effect (149). Remarkably, the growing development of proper nanosized materials that can protect biological ligands from degradation may represent the future golden age in cancer therapy to deliver PRR agonists directly into cancer cells, harnessing not only the role of PRRs in immune regulation but also their direct effect on tumors.

Notably, it has been reported that TLR9 agonists together with anti-PD-1 therapy can induce the neogenesis of tertiary lymphoid structures (TLSs) in mouse NSCLC tumors, which are composed of a well-organized lymphoid framework including B cells, DCs, and T cells in close contact adjacent within the TME (150). TLSs function as tumor-adjacent lymphoid sites for the priming and maintenance of adaptive humoral and cell-mediated responses against tumor antigens (151). TLS establishment in the TME correlates with long-term survival and a better response to immunotherapy in multiple human solid cancers (152, 153). TLR agonists in combination with ICIs can altogether sculpt the TME toward an antitumor milieu, thus enhancing ICI efficacy (**Figure 3**).

Furthermore, new technologies such as transcriptomic profiling, which have recently emerged in the field of precision medicine, can provide opportunities to gain insight into the complexity of the TME, where PRR plays a crucial role. Recently, an accessible multiomics platform that is able to classify and visualize the whole tumor composition, integrating transcriptomic and genomic data, has been developed. Their transcriptomic analysis of more than 10,000 cancer patients portrayed four distinct TME subtypes conserved across 20 different cancers, which correlate with patient response to immunotherapy in multiple cancers (154). More efforts should be put into unpuzzling the PRR complexity in immune cells to unveil the arrangement of how immune cells choose to open or close their “gates” of environmental recognition in response to stimuli in pathological contexts.

Collectively, MDSC PRRs have a key role in the TME-modulating process, and growing evidence on their regulatory proinflammatory and antitumor mechanisms combined with the development of novel analytic strategies, such as spatial transcriptomic analysis and precise refinement of the timing and method of administration of drug therapies based on PRRs, fuels promising anticancer therapeutic applications.

## Author contributions

All authors listed have made a substantial, direct, and intellectual contribution to the work and approved it for publication.
